# Increased Circulating T Follicular Helper Cells Are Inhibited by Rituximab in Neuromyelitis Optica Spectrum Disorder

**DOI:** 10.3389/fneur.2017.00104

**Published:** 2017-03-15

**Authors:** Cong Zhao, Hong-Zeng Li, Dai-Di Zhao, Chao Ma, Fang Wu, Ya-Nan Bai, Min Zhang, Zhu-Yi Li, Jun Guo

**Affiliations:** ^1^Department of Neurology, Tangdu Hospital, Fourth Military Medical University, Xi’an, China; ^2^Department of Cardiology, Tangdu Hospital, Fourth Military Medical University, Xi’an, China; ^3^Department of Neurology, Xi’an Children’s Hospital, Xi’an, China

**Keywords:** neuromyelitis optica spectrum disorder, circulating T follicular helper cells, rituximab, B cells, interleukin-6

## Abstract

Neuromyelitis optica spectrum disorder (NMOSD) is a severe autoimmune disease of the central nervous system. The existence of autoantibody targeting aquaporin-4 (AQP4-Ab) indicates the involvement of humoral immunity in the pathogenesis of this disease. Rituximab (RTX), a monoclonal antibody against CD20, has been used to treat NMOSD by depleting circulating B cells and overall satisfactory outcome has been achieved. Although T follicular helper cells have been proved to regulate B cell activation and antibody production, the role of these cells in NMOSD and the impact of RTX treatment on these cells remain less understood. In this study, we found that frequencies of circulating T follicular helper (cTfh) cells and B cells together with the related cytokines, IL-21 and IL-6, were closely correlated with disease activity of NMOSD. Furthermore, B cell depletion with RTX treatment inhibited the expansion of cTfh cells, and these effects were achieved through eliminating IL-6-producing B cells and blocking the direct contact between cTfh cells and B cells. These findings imply the complicated cross talk between cTfh cells and B cells and may provide a novel therapeutic target for NMOSD.

## Introduction

Neuromyelitis optica spectrum disorder (NMOSD) is a rare inflammatory demyelinating disease of the central nervous system (CNS) characterized by recurrent attacks of optic neuritis and transverse myelitis (1). In the past decades, NMOSD was believed to be a variant of multiple sclerosis (MS) based on the overlapping clinical and magnetic resonance imaging phenotypes. The identification of an autoantibody targeting aquaporin-4 (AQP4-Ab) has differentiated NMOSD from MS as an independent disease entity (2). AQP4-Ab is positive in more than half of patients with NMOSD, and its pathogenic role has been well demonstrated (3–5). Other antibodies against myelin antigens have also been reported in NMOSD, such as anti-MOG (6) and anti-MBP (7). Although the concrete role of these autoantibodies has not been clearly identified, recent evidence strongly suggested that humoral immunity would contribute to the pathology of NMOSD (8).

T follicular helper (Tfh) cells, as a newly defined CD4^+^ T cell subset, are critical for B cell activation and differentiation (9, 10). Tfh cells constitutively express the chemokine receptor CXCR5, which allows them to migrate into germinal center (GC) (11) and provide costimulation signals, such as CD40L and IL-21, to B cells (12). Recently, circulating Tfh (cTfh) cells were identified to be the peripheral subsets of their GC counterparts (13). And an increased frequency of cTfh cells was observed in multiple antibody-mediated diseases (14–17).

Rituximab (RTX), which depletes circulating CD20^+^ B cells, has emerged as the first-line immunosuppressant for treating NMOSD, though the precise mechanism still remains uncovered (18). In patients with MS, RTX treatment resulted in a significant decline of CNS-infiltrated T cells, which suggested this agent may also modulate T cell immune response besides depleting B cells (19). However in NMOSD, whether RTX exerts its therapeutic potential by regulating T cells, especially cTfh cells, still remains unclear.

In this study, we found that frequencies of cTfh cells and circulating B cells together with the related molecules were closely associated with the disease activity of NMOSD. Next, we firstly demonstrated that B cell depletion with RTX reduced the frequency of cTfh cells through ablation of IL-6 signaling and blockade of direct B–cTfh cell contact. The results strongly suggest the existence of B–cTfh cell interaction in NMOSD, which might provide a possible therapeutic target for this disease.

## Patients and Methods

### Study Population

We enrolled patients with NMOSD from June 2015 to March 2016 who fulfilled the 2015 revised NMOSD diagnostic criteria (20) in our department. Qualitative serum AQP4 antibody (AQP4-Ab) assay was done by cell-based indirect immunofluorescence [EUROIMMUN Medical Diagnostics (China) Co., Ltd.]. Patients with new neurologic symptoms and signs or deterioration of residual disability lasting for at least 24 h with new lesions on MRI were determined as relapse. Patients with a stable clinical status for at least 30 days since the last relapse were considered as remission and enrolled in this study. Meanwhile, gender- and age-matched healthy volunteers were included as controls (healthy controls). The study was approved by the Tangdu Hospital Ethical Review Board of Fourth Military Medical University, and written informed consent was obtained from all the subjects.

### Sampling and Treatment

Blood samples were drawn from all the patients and HCs to collect peripheral blood mononuclear cells (PBMCs) and plasma for detecting cell frequencies and cytokine concentrations, respectively. Rituximab (RTX) treatment was carried out in the patients based on clinical status and patient’s preference. Intravenous infusion of RTX at a fixed dose of 100 mg was performed once weekly for three consecutive weeks, as previously described (21). Blood samples were collected again 1 month after RTX treatment. Meanwhile, plasma AQP4-Ab levels were measured, respectively, before and 1 month after RTX treatment in seropositive patients.

### Flow Cytometry

Peripheral blood mononuclear cells were isolated by density gradient centrifugation as previously reported (17). After washed twice with phosphate-buffered saline (PBS), PBMCs were incubated with the following fluorochrome-conjugated monoclonal Abs: FITC-CD3, PerCP-Cy5.5-CD4, APC-CXCR5, PE-PD-1, PE-CD19, and relevant isotype controls (Biolegend, San Diego, CA, USA). After staining at 4°C for 30 min, PBMCs were washed twice with PBS containing 2% fetal bovine serum and then measured on a BD FACS Calibur instrument. cTfh cells were defined as CD3^+^CD4^+^CXCR5^+^PD-1^+^. Data were analyzed using the FlowJo 7.6 software.

### Detection of Cytokines and AQP4-Ab

Plasma levels of IL-21, IL-6, and IL-10 were measured with enzyme-linked immunosorbent assay (ELISA) (Biolegend for IL-21, and Dakewe for IL-6 and IL-10) according to the manufacturers’ instructions. For seropositive patients, plasma AQP4-Ab levels were also measured by ELISA (Cusabio, Wuhan, China).

### Cell Sorting and Culturing

Circulating CD4^+^ T cells and CD19^+^ B cells were isolated from PBMCs by using specific magnetic beads (Miltenyi Biotec, Bergisch, Germany). To explore the role of B cells in maintenance of cTfh cells and the underlying mechanisms, 2 × 10^5^ of whole or B cell-depleted PBMCs were stimulated with 1 μg/ml plate-bounded anti-CD3 (Biolegend, San Diego, CA, USA) and 1 μg/ml soluble anti-CD28 (Biolegend, San Diego, CA, USA) for 72 h, in presence or absence of IL-6-neutralizing mAb (5 μg/ml; Biolegend, San Diego, CA, USA), in a 96-well flat-bottom plate. In the transwell culture system, 2 × 10^5^ of B cell-depleted PBMCs were cultured and stimulated with anti-CD3/CD28 in the lower chamber and autologous B cells were seeded into the inner chamber of 0.4 μm pore size (Merck Millipore, Billerica, MA, USA). Each coculture of cells was carried out in triplicate in the X-VIVO serum-free medium (Lonza, Basel, Switzerland) containing 1% penicillin/streptomycin (Sigma, St. Louis, MO, USA).

### Coculture of Circulating CD4^+^ T Cells and CD19^+^ B Cells

To determine the potency of B cells in the maintenance of cTfh cells, 2 × 10^5^ of CD4^+^ T cells were cocultured with autologous CD19^+^ B cells at different ratios (as indicated in Figure 5) for 72h in the presence of anti-CD3 (1 μg/ml) and anti-CD28 (1 μg/ml). Frequencies of cTfh cells were measured with the markers CD3, CD4, CXCR5, and PD-1 on a BD FACS Calibur instrument.

### Statistical Analysis

Quantitative data are shown as means ± SEM, and categorical data are presented as number with percentage. Statistical analysis was performed using the SPSS19.0 software. Demographic and clinical characteristics among the relapsing patients, remitting patients, and HCs were compared with Fisher’s exact test (gender, AQP4-Ab positive) and ANOVA (age, duration of disease). Multiple comparisons among the different groups were carried out with ANOVA for normally distributed data and with Kruskal–Wallis *H* non-parametric test for non-normally distributed data. Comparison between pre- and post-RTX treatment was performed with Wilcoxon matched-pairs signed-rank test. Pearson’s correlation test was used to measure the possible relationship between two variables of interest. A *P* value of less than 0.05 was considered as statistically significant.

## Results

### Demographic and Clinical Characteristics of Patients with NMOSD and HCs

A total of 31 patients and 18 gender- and age-matched HCs were enrolled in this study, where NMOSD patients consisted of 15 relapsing and 16 remitting individuals. There were no difference found in the gender ratio and mean age among the relapsing patients, remitting patients, and HCs. A predominance of female was observed in both relapsing (93.3%) and remitting patients (93.8%) with a similar mean duration of disease (3.19 vs 4.00 months). Serum AQP4-Ab was positive in 24/31 (77.4%) patients. There were 11/15 (73.3%) relapsing patients and 13/16 (81.3%) remitting patients, respectively, positive for AQP4-Ab, with no significant intergroup difference seen (Table 1).

**Table 1:** **Demographic and clinical characteristics of patients with NMOSD and HCs**.

	NMOSD		HCs	*P* value
	Relapse	Remission		
*n*	15	16	18	
Female, no. (%)	14 (93.3)	15 (93.8)	16 (88.9)	1.000
Age (years)	42.53 ± 3.16	47.19 ± 2.63	41.61 ± 2.81	0.343
Duration of disease (months)	3.19 ± 0.72	4.00 ± 0.79	NA	0.457
AQP4-Ab positive, no. (%)	11 (73.3)	13 (81.3)	NA	0.685

*Ab, antibody; AQP4, aquaporin-4; NMOSD, neuromyelitis optica spectrum disorders; HCs, healthy controls; NA, not applicable. Quantitative variables are presented as means ± SEM and categorical variables as number with percentages. Comparisons among the relapsing patients, remitting patients, and HCs were analyzed by ANOVA (age, duration of disease) and Fisher’s exact test (gender ratio, AQP4-Ab positive). A *P* value of <0.05 was assumed as statistically significant*.

### Frequencies of cTfh Cells and B Cells Correlate with Disease Activity of NMOSD

**Figure 1:**
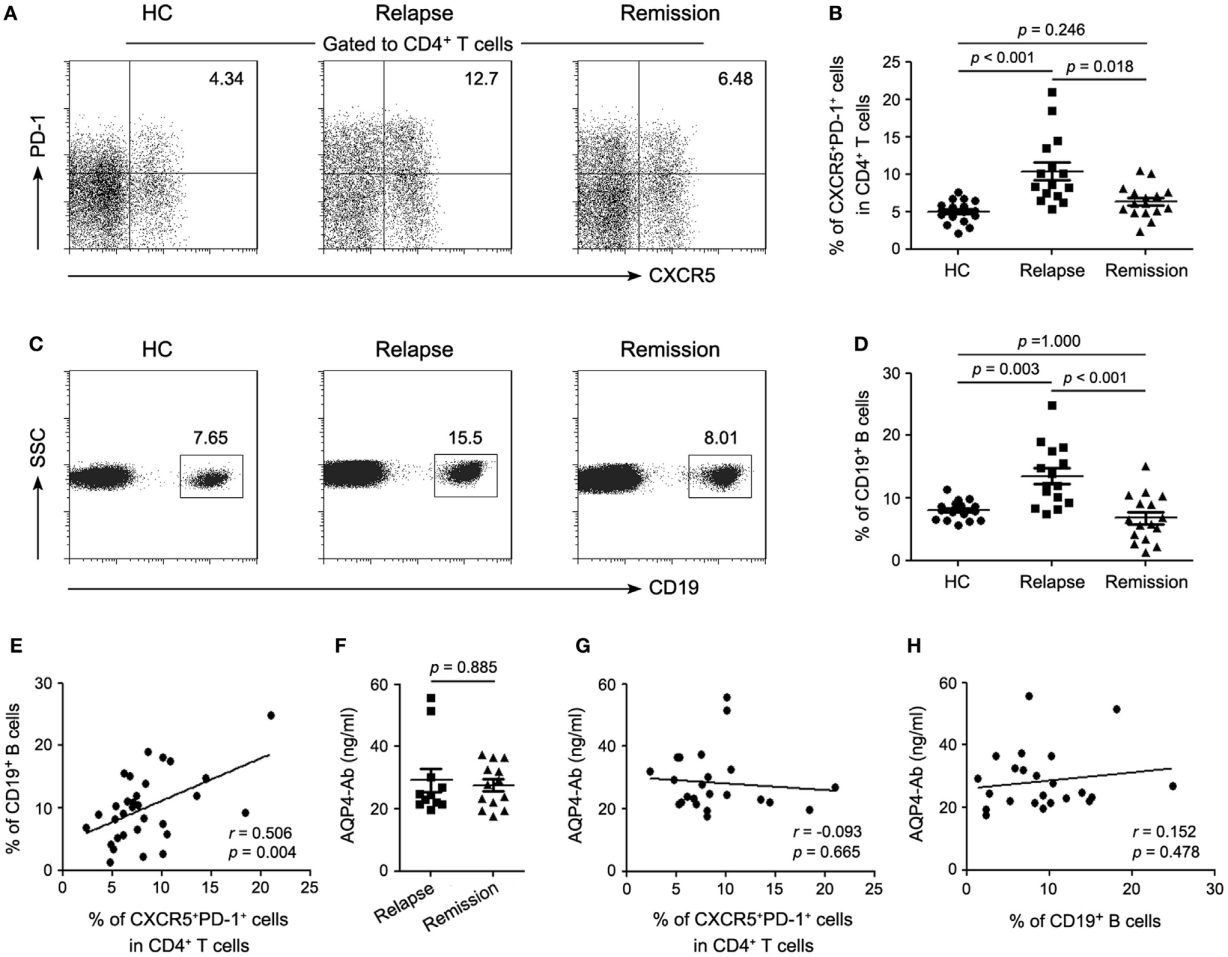
**Frequencies of circulating T follicular helper (cTfh) cells and circulating CD19^+^ B cells correlate with disease activity of neuromyelitis optica spectrum disorder (NMOSD)**. **(A)** Representative flow cytometry plots showing the frequency of CD4^+^CXCR5^+^PD-1^+^ cTfh cells in healthy controls (HCs), relapsing and remitting patients with NMOSD. **(B)** Comparison of the frequency of cTfh cells. **(C)** Representative flow cytometry plots gated on lymphocytes showing frequency of circulating CD19^+^ B cells. **(D)** Comparison of the frequency of circulating CD19^+^ B cells. **(E)** Correlation between frequencies of cTfh cells and circulating CD19^+^ B cells. **(F)** Comparison of plasma AQP4-Ab level between the relapsing and remitting seropositive patients with NMOSD. **(G)** Correlation between the frequency of cTfh cells and plasma AQP4-Ab level. **(H)** Correlation between the frequency of circulating CD19^+^ B cells and plasma AQP4-Ab level. Each symbol, including solid circle, square, and triangle, represents one subject’s result. Horizontal lines in panels **(B,D,F)** illustrate the mean frequencies or levels with SEM. *P* values are shown.

Flow cytometry results showed that the frequency of cTfh cells in the relapsing patients with NMOSD was significantly higher than those in the remitting patients and HCs, while no difference existed between the latter groups, suggesting a correlation with disease activity of NMOSD (Figures 1A,B). Moreover, there was a similar tendency on the change of frequency of peripheral B cells (Figures 1C,D). A positive correlation was found between frequencies of cTfh cells and B cells among the patients with NMOSD (Figure 1E). Subsequently, we detected plasma AQP4-Ab levels in seropositive patients. No difference was found between the relapsing and remitting patients (Figure 1F). In addition, frequencies of both cTfh cells and B cells had no correlations with plasma AQP4-Ab level (Figures 1G,H).

### Cytokines Concentration in Patients with NMOSD and HCs

**Figure 2:**
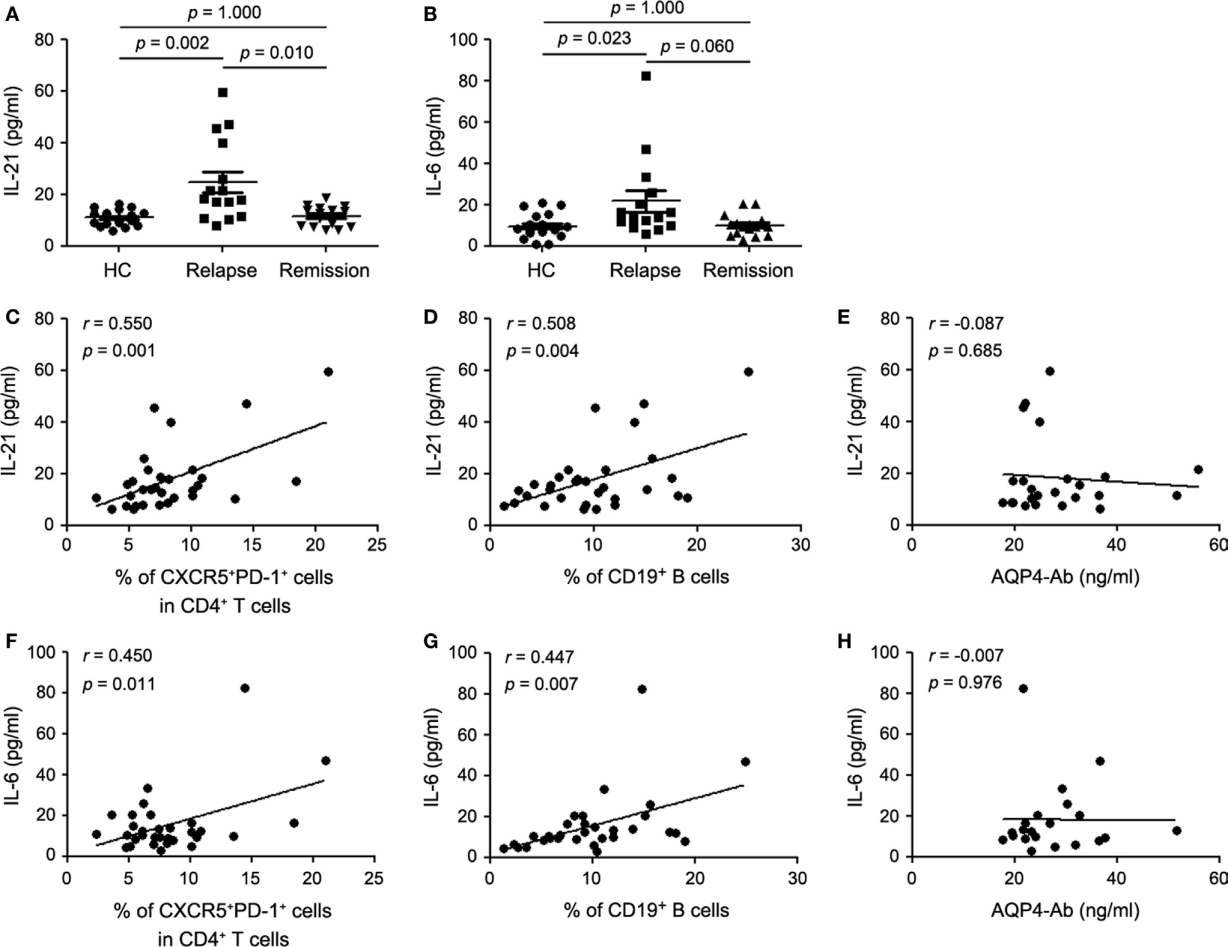
**Plasma cytokine levels in healthy controls (HCs), the relapsing and remitting patients with neuromyelitis optica spectrum disorder (NMOSD)**. **(A)** Comparison of plasma IL-21 level. **(B)** Comparison of plasma IL-6 level. **(C)** Correlation between plasma IL-21 level and the frequency of circulating T follicular helper (cTfh) cells in all enrolled patients with NMOSD. **(D)** Correlation between plasma IL-21 level and the frequency of circulating CD19^+^ B cells in all enrolled patients with NMOSD. **(E)** Correlation between plasma IL-21 level and AQP4-Ab in seropositive patients with NMOSD. **(F)** Correlation between plasma IL-6 level and the frequency of cTfh cells in all enrolled patients with NMOSD. **(G)** Correlation between plasma IL-6 level and the frequency of circulating CD19^+^ B cells in all enrolled patients with NMOSD. **(H)** Correlation between plasma level of IL-6 AQP4-Ab in seropositive patients with NMOSD. Each symbol represents one subject’s result. Horizontal lines in panel **(C)** illustrate the mean frequencies with SEM. *P* values are shown.

Given the fact that IL-21 and IL-6 are pivotal regulators of humoral immune response and play a crucial role in Tfh cell differentiation, we evaluated the plasma levels of IL-21 and IL-6 by ELISA. There was a significant increase of plasma IL-21 and IL-6 levels in the relapsing patients with NMOSD compared with the remitting patients and HCs (Figures 2A,B), which was consistent with the changes of cTfh cells and B cells. Meanwhile, plasma level of IL-10, an anti-inflammatory cytokine, was also detected and a significant increase was found in the relapsing patients. Although there was a tendency of higher IL-10 levels in plasma of remitting patients than HCs, no significant difference was observed (Figure S1A in Supplementary Material). Correlation analysis revealed that plasma IL-21 level positively correlated with frequencies of both cTfh cells and B cells (Figures 2C,D). The same phenomenon was observed for IL-6 (Figures 2F,G) but not for IL-10 (Figures S1B,C in Supplementary Material). In addition, no correlation was found between plasma levels of IL-21, IL-6, and IL-10, respectively, and plasma AQP4-Ab levels (Figures 2E,H; Figure S1D in Supplementary Material).

### RTX Treatment Reduced cTfh Cells in Patients with NMOSD

**Figure 3:**
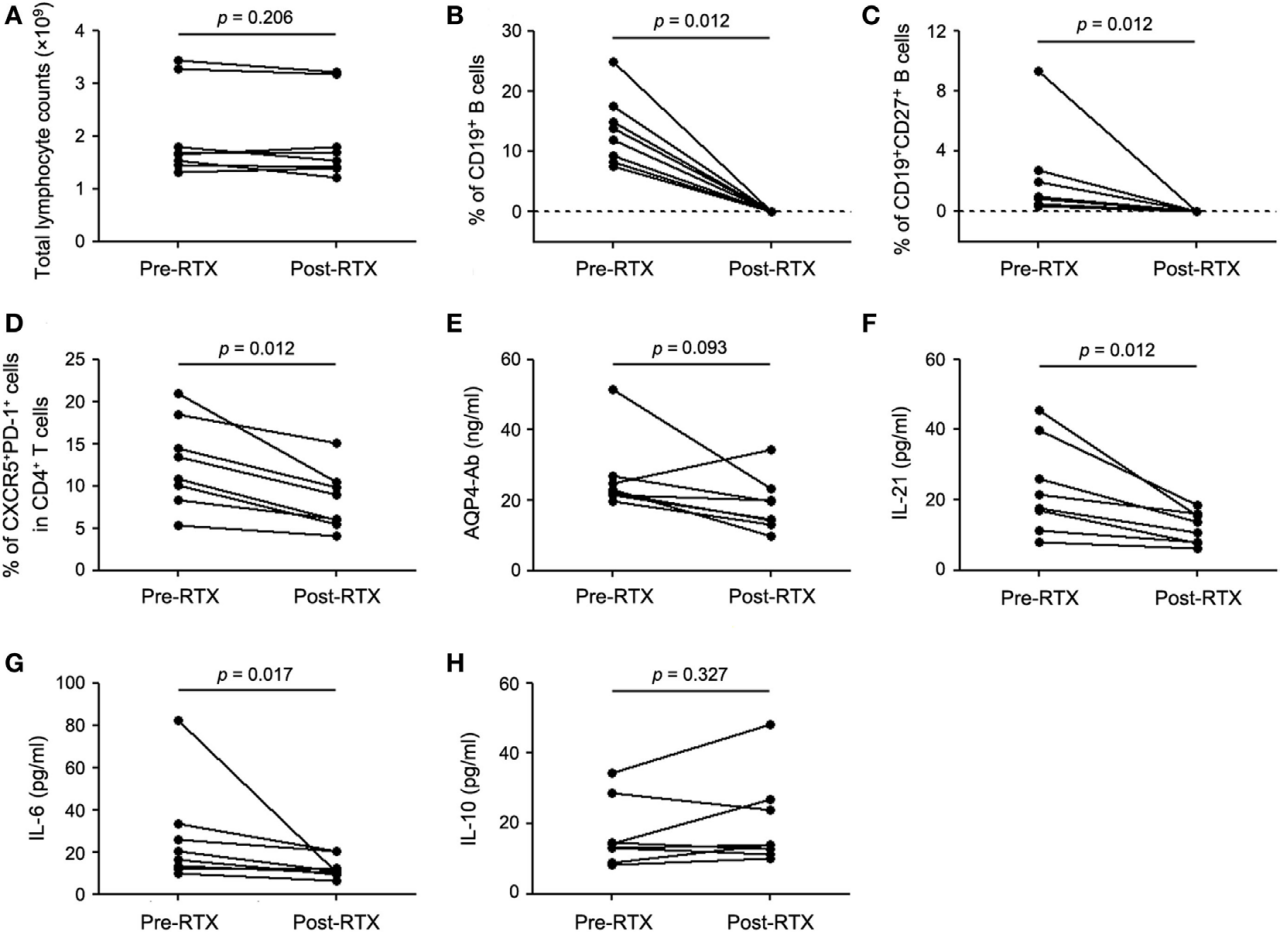
**B-cell depletion with RTX decreases the frequency of circulating T follicular helper (cTfh) cells and plasma levels of IL-21 and IL-6**. **(A)** Comparison of total lymphocyte counts between pre- and post-RTX treatment. **(B)** Comparison of the frequency of circulating CD19^+^ B cells. **(C)** Comparison of the frequency of CD19^+^CD27^+^ memory B cells. **(D)** Comparison of the frequency of cTfh cells. **(E)** Comparison of plasma AQP4-Ab level. **(F)** Comparison of plasma IL-21 level. **(G)** Comparison of plasma IL-6 level. **(H)** Comparison of plasma IL-10 level. Line represents the changes of the frequency of specific cells, plasma AQP4-Ab, and cytokine levels before and after RTX treatment. *P* values are shown.

RTX specifically depletes peripheral B cells and has been used as a first-line immunosuppressant for NMOSD. To further explore the possible effects of RTX on cTfh cells, eight seropositive patients with relapsing NMOSD enrolled in this study were treated with RTX in our center. The total lymphocyte counts in peripheral blood remained almost unchanged during RTX treatment (Figure 3A). All the patients responded well to RTX and circulating B cells were successfully depleted (Figures 3B,C; Table S1 in Supplementary Material). Furthermore, CD4^+^ T cells decreased after RTX treatment (Figure S2 in Supplementary Material). Notably, the frequency of cTfh cells was significantly declined with RTX treatment (Figure 3D). Furthermore, a decreased tendency of plasma AQP4-Ab level was observed after RTX treatment but with no statistical significance (Figure 3E). Consistent with the change of cTfh cells, plasma levels of IL-21 and IL-6 were obviously decreased with RTX treatment (Figures 3F,G), while no significant alteration of plasma IL-10 level was observed between pre- and post-RTX treatment (Figure 3H). Meanwhile, we did *ex vivo* experiments which showed that B cells depletion could significantly reduce the frequency of IL-21-secreting CD4^+^ T cells (i.e., Tfh cells). And the frequency of IL-17-secreting cells (i.e., Th17 cells) slightly decreased, whereas the frequency of the other CD4^+^ T cell subsets was almost unchanged. This finding suggested that Tfh cells may be more sensitive to B cells depletion (Figure S3 in Supplementary Material).

### Both IL-6 and Direct B/cTfh Contact Were Essential for the Maintenance of cTfh Cells

**Figure 4:**
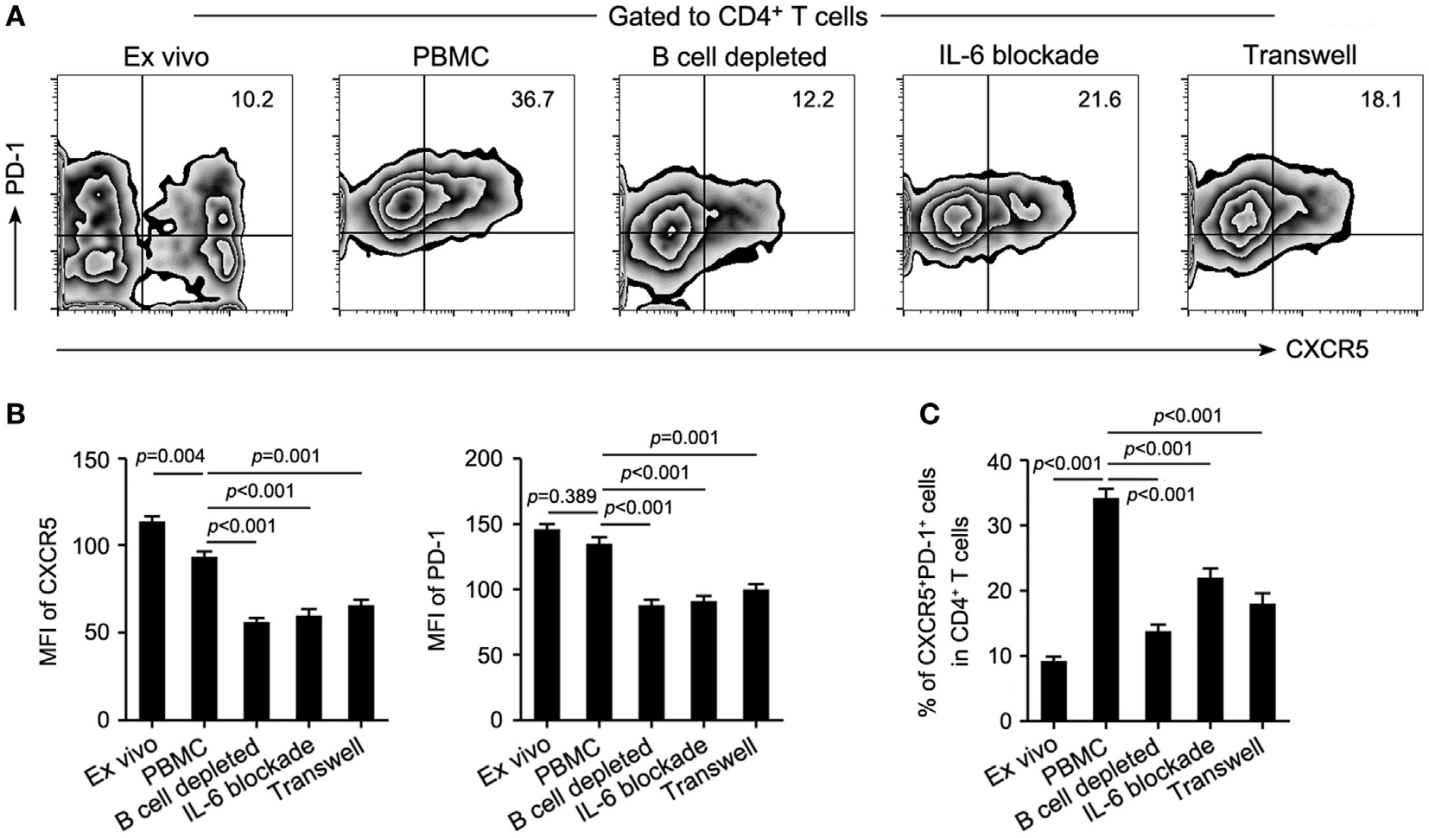
**Both IL-6 production and direct cell–cell contact are necessary for the maintenance of circulating T follicular helper (cTfh) cells by B cells**. B cell-depleted and whole peripheral blood mononuclear cells (PBMCs) from patients with neuromyelitis optica spectrum disorder were cultured upon stimulation with anti-CD3/CD28 for 72 h (*n* = 4), then the frequency of cTfh cells in the coculture system was measured. **(A)** Representative flow cytometry plots showing the frequency of T follicular helper cells of indicated cultured conditions. **(B)** Mean fluorescence intensity (MFI) of CXCR5 (left) and PD-1 (right) of CD4^+^CXCR5^+^PD-1^+^ T cells in each group. **(C)** Comparison of the frequency of cTfh cells in panel **(A)**. *P* values are shown.

**Figure 5:**
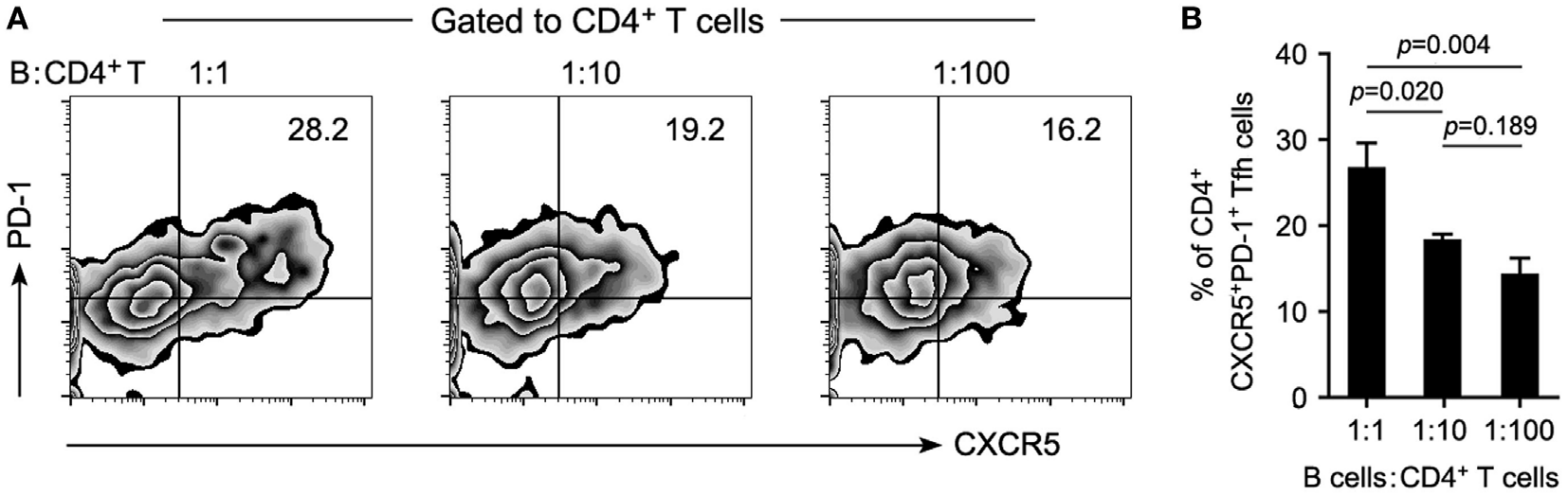
**Reduced B cells attenuate the maintenance of T follicular helper (Tfh) cells in a ratio-dependent manner**. Circulating CD19^+^ B cells were cultured with CD4^+^ T cells on different ratios of 1:1, 1:10, and 1:100 and stimulated with anti-CD3/CD28 for 72 h. Maintenance of Tfh cells by B cells was estimated by flow cytometry. **(A)** Representative flow cytometry plots showing the frequency of Tfh cells. **(B)** Cumulative data showing the frequency of Tfh cells (*n* = 4 for each group). A reduced ratio of B cells in the coculture system was accompanied by the gradually attenuated maintenance of Tfh cells. *P* values are shown. Representative data are from three independent experiments.

It is known that Tfh cells promote B cell proliferation, activation, and differentiation. However, B-cell depletion with RTX in this study significantly decreased the frequency of cTfh cells. This suggested that B cells might contribute to the maintenance of cTfh cells reciprocally. To verify this hypothesis, PBMCs from patients with NMOSD were cultured *ex vivo* upon stimulation with anti-CD3/CD28, in parallel, B cell-depleted PBMCs were cultured to mimic RTX treatment. We observed a significantly reduced frequency of cTfh cells in B cell-depleted group (Figures 4A,C). To verify whether B cells directly affect the frequency of cTfh cells, we cocultured CD4^+^ T cells with B cells in different ratios under anti-CD3/CD28 stimulation and found that cTfh cells were maintained by B cells in a ratio-dependent manner (Figure 5), which suggested the direct supporting function of B cells.

In addition to the rise of plasma IL-6 level in relapsing patients (Figure 2B) and its positive correlation with circulating B cells in NMOSD (Figure 2G), we further observed an elevated mRNA level of IL-6 in B cells from the relapsing patients (Figure S4 in Supplementary Material), suggesting that B cells might maintain cTfh population through secretion of IL-6. As expected, IL-6 blockade indeed obviously reduced the frequency of cTfh cells in the culture system of PBMCs even though B cells were not depleted (Figures 4A,C). Besides, lack of direct contact between B cells and cTfh cells in a transwell culture system also led to a significant reduction of the frequency of cTfh cells (Figures 4A,C). Furthermore, all of the B-cell depletion, IL-6 blockade, and transwell experiments significantly reduced the protein level of CXCR5 and PD-1 on cTfh cells, as shown by the mean fluorescence intensity (MFI) in Figure 4B.

## Discussion

T follicular helper cells have been identified as the most potent regulator of humoral immunity. Given the location of Tfh cells in the GC of secondary lymphoid organs, it is extremely hard to be obtained from patients routinely. Morita and colleagues have demonstrated that circulating CD4^+^CXCR5^+^ T cells appeared to be the memory subset of GC Tfh pool and had the same capacity to regulate B cells (13), which facilitated researchers to explore the role of these cells in human diseases. IL-21 and IL-6 are two major cytokines in regulating GC response, including Tfh cell proliferation and differentiation, B cell activation, and antibody production (22). A lack of both IL-6 and IL-21 fails to induce Tfh cell-dependent immune response; moreover, activated Tfh cells can produce a considerable amount of IL-21 (22). In accordance with a previous report (23), herein, we found that the frequency of cTfh cells as well as plasma levels of IL-21 and IL-6 were significantly upregulated in relapsing patients with NMOSD but not in remitting patients, compared with those in HCs. Both IL-21 and IL-6 were positively correlated with the frequency of cTfh cells. These results suggested that cTfh cells might be involved in the pathogenesis of NMOSD and the frequency of cTfh cells, together with plasma levels of IL-21 and IL-6, could be used as biomarkers for monitoring disease activity of NMOSD.

T follicular helper cells play the central role in helping B cells activation and differentiation, which is protective against infection. However, overactivity of Tfh response could manifest as many immune-related disorders, such as autoimmunity (24). The frequency of Tfh cells, together with the titer of autoantibodies, was found to elevate in many autoimmune disorders, both in animal models and human (14–17, 25–28). In this study, although no correlation between plasma AQP4-Ab level and disease activity of NMOSD was observed, the frequencies of circulating B cells and cTfh cells were synchronously upregulated in relapsing patients and positively correlated with each other. However, in our study, no correlation of AQP4-Ab titers with disease activity is observed, which is consistent with previous studies (29). This implies that cTfh cells might promote NMOSD by activating B cells to secrete cytokines other than producing antibodies, a hypothesis that needs further investigation.

B cells might contribute to autoimmunity *via* multiple ways, including antigen presentation, cytokine secretion, and antibody production. Depleting B cells with RTX is an effective approach to treat autoimmune disease, though the underlying mechanisms still remain debatable. In this study, circulating B cells were successfully depleted even by a reduced dose of RTX, which was consistent with a previous study (21). However, RTX treatment could only moderately reduce plasma level of AQP4-Ab, probably attributed to the fact that RTX removed CD20^+^ B cells but spared CD20^−^ antibody-producing plasma cells. Likewise, several studies reported that RTX treatment was effective for NMOSD (29) and other autoimmune diseases even in patients who showed no decline of autoantibody titers (30, 31), or the benefit of RTX treatment usually preceded the drops of antibody levels (30). All the findings suggested that B cells could contribute to the pathology through mechanisms other than antibody production.

Interestingly, we found that CD4^+^ T cells declined after RTX treatment. Given the multiple effects of B cells on T cell function, we think that RTX treatment may indeed have a non-specific effect on the frequency of the entire CD4^+^ T cells. Due to the fact that Tfh cells are the true activator of humoral immunity, our study focused on the impact of RTX treatment on cTfh cells and its related mechanism. We found that RTX treatment also significantly reduced the frequency of cTfh cells in patients with NMOSD. Other studies have also reported this phenomenon in some other autoimmune diseases (32, 33), but they did not elaborate the concrete mechanisms involved. We then further tried to explore the potential mechanisms *via* which B cells regulate Tfh response. A candidate factor may be IL-6, since it has been proved to play a vital role in the differentiation of Tfh cells (34). A study showed that B cells could secret an abundance of IL-6 and exhibited pathogenic effects in experimental autoimmune encephalomyelitis, an animal model for MS (35). Furthermore, circulating plasmablasts could induce the differentiation of Tfh cells *via* producing IL-6 in patients with rheumatoid arthritis, and IL-6 blockade reduced the population of Tfh cells (36). Indeed, we observed a significant increase of plasma IL-6 level in patients with NMOSD and further demonstrated an elevated mRNA level of IL-6 in B cells, which suggested that B cells might be an important origin of IL-6. B cell depletion, both *ex vivo* and *in vivo*, decreased cTfh cells from patients with NMOSD, and this effect was achieved in a ratio-dependent manner *ex vivo*. Meanwhile, plasma IL-6 level was markedly decreased with RTX treatment in parallel with the change of frequency of B cells. Further study showed that IL-6 was required for the maintenance of cTfh cells by B cells, since blockade of IL-6 reduced the frequency of cTfh cells in the coculture system. Moreover, the intimate contact between B cells and cTfh cells was also essential for the survival of cTfh cells as proved by a transwell culture system. This contact-dependent maintenance of Tfh cells by B cells in this disease could be achieved by the crosslink of ICOS–ICOSL (37), OX40–OX40L (38), and MHC-II–TCR (39, 40) on the surface of these cells. But the central molecule involved in the cell–cell contact in this disease still needs a further research. Previous studies have reported that IL-6 and these costimulatory molecules were able to stimulate the expression of CXCR5 and PD-1 (12, 41), and we also observed the reduction of MFI on CXCR5 and PD-1 in the *ex vivo* experiments. This suggested that the decrease of cTfh frequency might be achieved gradually by reducing the expression level of CXCR5 and PD-1. Taken together, our data raised a potential positive feedback loop between B and cTfh cells, namely, that circulating B cells maintain cTfh cells, and cTfh cells in turn activate B cells to persistently produce more IL-6 and costimulation molecules. This loop could be interrupted by RTX treatment, which revealed a new mechanism of RTX in treating NMOSD.

In conclusion, this study demonstrated that cTfh cells, circulating B cells, and associated molecules might play an important role in the pathogenesis of NMOSD. B cell depletion with RTX could reduce the frequency of cTfh cells through eliminating IL-6 signaling and blocking the direct B–cTfh cell contact. All the findings may provide an insight into the complicated cross talk between B cells and cTfh cells in NMOSD.

## Ethics Statement

This study was carried out in accordance with the recommendations of “the Biomedical Research Guideline involving Human Participants, National Health and Family Planning Commission of China” with written informed consent from all subjects. All subjects gave written informed consent in accordance with the Declaration of Helsinki. The protocol was approved by the “Tangdu Hospital Ethical Review Board of Fourth Military Medical University.”

## Author Contributions

JG and Z-YL designed the research. CZ performed the flow cytometry, cell cultures, and ELISA and drafted the manuscript. H-ZL and D-DZ took care of and followed up the patients, and helped revise the manuscript. FW and CM did the cell sorting. Y-NB and MZ performed the quantitative PCR. All of the authors read and approved the publication.

## Conflict of Interest Statement

The authors declare that the research was conducted in the absence of any commercial or financial relationships that could be construed as a potential conflict of interest.
