# Supplementary material for: Increased Circulating T Follicular Helper Cells Are Inhibited by Rituximab in Neuromyelitis Optica Spectrum Disorder
Source: Front Neurol. 2017 Mar 15;8:104. doi: 10.3389/fneur.2017.00104 (PMC5350120; doi:10.3389/fneur.2017.00104)
Supplement: Supplementary file 1 [file Data_Sheet_1.doc]

**Supplemental Data**

**Increased circulating T follicular helper cells are inhibited by Rituximab in neuromyelitis optica spectrum disorder**

Cong Zhao^a,1^, Hong-Zeng Li^a,1^, Dai-Di Zhao^a,1^, Chao Ma^b^, Fang Wu^a,c^, Ya-Nan Bai^a^, Min Zhang^a^, Zhu-Yi Li^a^*, Jun Guo^a^*

^a^Department of Neurology, Tangdu Hospital, Fourth Military Medical University, Xi’an Shaanxi, 710038, China

^b^Department of Cardiology, Tangdu Hospital, Fourth Military Medical University, Xi’an Shaanxi, 710038, China

^c^Department of Neurology, Xi’an Children’s Hospital, Xi’an Shaanxi, 710003, China

*To whom correspondence should be addressed

Jun Guo, Department of Neurology, Tangdu Hospital, Fourth Military Medical University, Xi’an Shaanxi, 710038, China.

Tel: +86-29-84717483; Fax: +86-29-83552982; E-mail: [guojun_81@163.com](mailto:guojun_81@163.com)

Zhu-Yi Li, Department of Neurology, Tangdu Hospital, Fourth Military Medical University, Xi’an Shaanxi, 710038, China.

Tel: +86-29-84777443; Fax: +86-29-83552982; E-mail: [lizhuyi@fmmu.edu.cn](mailto:lizhuyi@fmmu.edu.cn)

**SUPPLEMENTAL METHODS**

**Quantitative real-time PCR analysis of IL-6 mRNA in B cells**

Total RNA of B cells was extracted from relapsing patients with NMOSD and HCs using TRIzol reagent (Invitrogen, Carlsbad, CA, USA), and the quality of RNA was evaluated with a Nanodrop spectrophotometer (ThermoFisher Scientific, Waltham, MA, USA). cDNA was synthesized with SuperScript III first-strand synthesis system (ThermoFisher Scientific, Waltham, MA, USA). For quantitative PCR analysis, the mRNA level of IL-6 was assessed on a Bio-rad PTC 200 system using a SYBR Green real-time kit (Takara, Tokyo, Japan). Each sample was run in triplicate and data were analyzed through the 2^-ΔΔCt^ method and normalized to the mRNA level of GAPDH. The primer sequences were listed as follows: IL-6: 5’-CAC ACA GAC AGC CAC TCA CC-3’ (forward) and 5’-TTT TCT GCC AGT GCC TCT TT-3’ (reverse); GAPDH: 5’-AAG GTG AAG GTC GGA GTC AA-3’ (forward) and 5’-TGG ACT CCA CGA CGT ACT CA-3’ (reverse).

***Ex vivo* B cell depletion and Intracellular cytokine staining**

To test the sensitivity of all CD4^+^ T cell subsets to RTX treatment, we performed *ex vivo* B cells depletion experiments, as previously described in the main text. 1×10^6^ of PBMCs, was stimulated with phorbol 12-myristate 13-acetate (PMA, 1 mg/mL), ionomycin (IONO, 0.25 mg/mL) and 10 μg/ml brefeldin A (BD Biosciences, San Jose, CA) for 5 h. 1×10^6^ of whole or B cell-depleted PBMCs were stimulated with 1 μg/ml plate-bounded anti-CD3 (Biolegend, San Diego, CA, USA) and 1 μg/ml soluble anti-CD28 (Biolegend, San Diego, CA, USA) for 72 h, followed by treatment with PMA, IONO and brefeldin A for 5 h. After stimulation, cells were stained with APC-CD3 and PerCP-Cy5.5-CD4. After cell surface staining, cells were treated with Cytofix/Cytoperm kit (BD Biosciences, San Jose, CA) according to the manufacturer’s instruction. Then intracellular staining was performed using FITC-IFN-γ, FITC-IL-4, FITC-IL-17 and FITC-IL-21. Flow cytometry was carried out with a BD FACS Calibur instrument. Data ware analyzed using FlowJo7.6 software.

**SUPPLEMENTAL RESULTS**

**Supplemental Table 1.** Frequencies of total B cells and CD19^+^CD27^+^ memory B cells before and after RTX treatment

|  | **Pre-RTX** | **Post-RTX** | ***p* value** |
| --- | --- | --- | --- |
| CD19^+^ B cells | 13.490 ± 2.042 | 0.013 ± 0.003 | 0.012 |
| CD19^+^CD27^+^ memory B cells | 2.145 ± 1.065 | 0.006 ± 0.002 | 0.012 |

Frequencies of circulating B cell subsets in 8 patients with NMOSD receiving RTX treatment were included and expressed as means ± SEM (standard error of mean). Comparison between pre- and post-RTX treatment were performed by Wilcoxon matched-pairs signed-rank test, and a *P* value of less than 0.05 was considered as statistically significant.


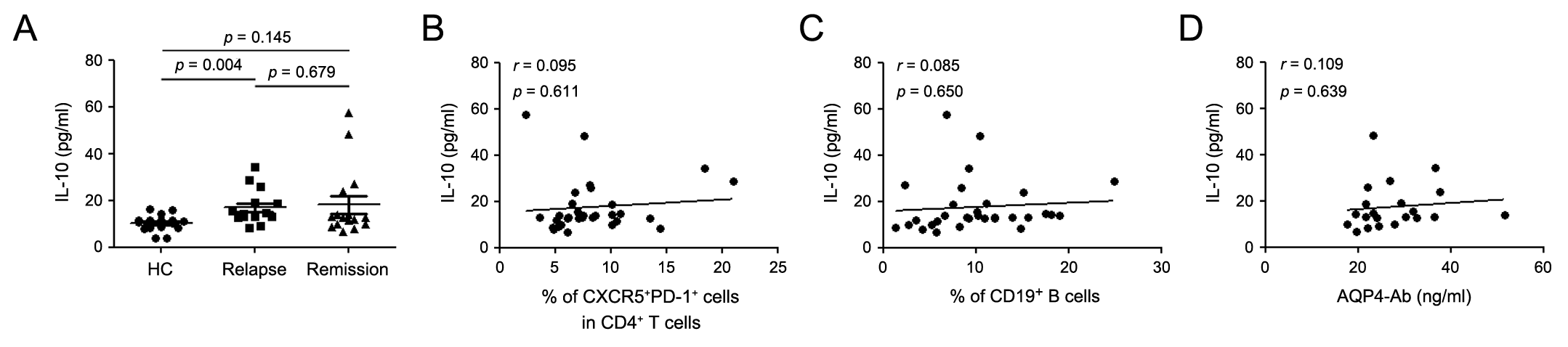


**Supplemental Figure 1. Plasma level of IL-10 in HCs, the relapsing and remitting patients with NMOSD.** (A) Comparison of plasma IL-10 level. (B) Correlation between plasma IL-10 level and the frequency of cTfh cells in all enrolled patients with NMOSD. (C) Correlation between plasma IL-10 level and the frequency of circulating B cells in all enrolled patients with NMOSD. (D) Correlation between plasma level of IL-10 and AQP4-Ab in seropositive patients with NMOSD. Each symbol represents one subject’s result. Horizontal lines in Figure A illustrate the mean frequencies with SEM. *P* values are shown.


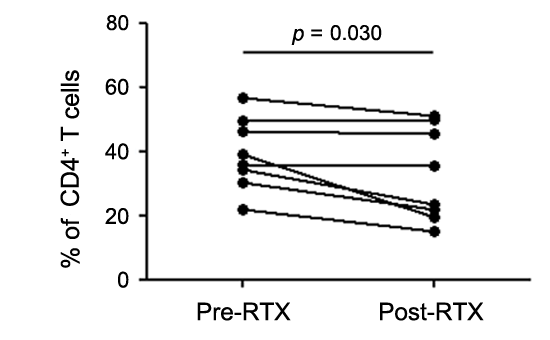


**Supplemental Figure 2. The frequency of CD4^+^ T cells in the patients with NMOSD decreased after RTX treatment.** Each symbol represents one subject’s result. The frequency of CD4+ T cells before and after RTX therapy in each patient are connected with solid line (n=8). Paired T test was used to perform statistical analysis. *P* value are shown.


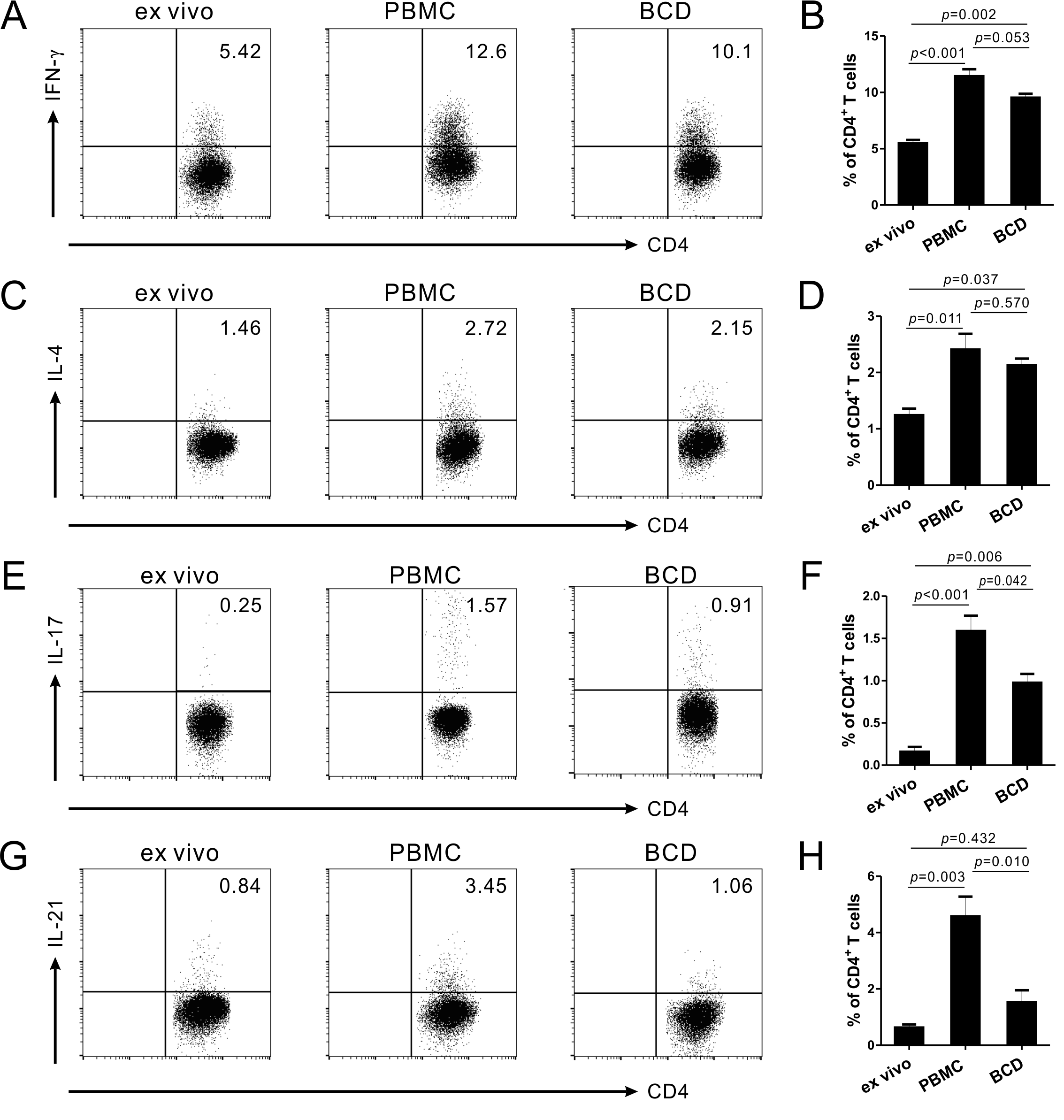


**Supplemental Figure 3.** **IL-21-secreting CD4^+^ T cells were more sensitive to B cell depletion compared to other CD4^+^ T cell subsets.** Representative flow cytometry plots showing the frequency of IFN-γ^+^ (A), IL-4^+^ (C), IL-17^+^ (E) and IL-21^+^ (G) CD4^+^ T cells of each group (B, D, F, and H). Comparison of the frequency of indicated cells (n=4). *P* values are shown.


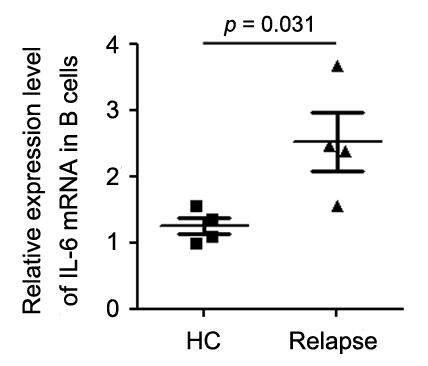


**Supplemental Figure 4. Expression of IL-6 mRNA in B cells is increased in relapsing patients with NMOSD.** IL-6 mRNA levels in B cells from the relapsing patients with NMOSD and HCs were detected by quantitative RT-PCR. Expression of IL-6 mRNA in B cells was significantly higher in relapsing patients with NMOSD than that in HCs (*n* = 4 for each group). *P* values are shown. Representative data are from three independent experiments.
